# Biochemical Characterization of Liver Oil of* Echinorhinus brucus* (Bramble Shark) and Its Cytotoxic Evaluation on Neuroblastoma Cell Lines (SHSY-5Y)

**DOI:** 10.1155/2016/6294030

**Published:** 2016-05-31

**Authors:** Vishnu Venugopal, Ajeeshkumar Kizhakkepurath Kumaran, Niladri Sekhar Chatterjee, Suvanish Kumar, Shyni Kavilakath, Jayarani Ramachandran Nair, Suseela Mathew

**Affiliations:** ^1^Central Institute of Fisheries Technology (CIFT), ICAR, Matsyapuri P.O., Kerala 682029, India; ^2^National Institute of Technology (NIT), Calicut, Kerala 673601, India

## Abstract

The objective of the present study was to characterize the liver oil extracted from the deep sea shark,* Echinorhinus brucus*, caught from Central Indian Ocean and to evaluate its cytotoxic effect on neuroblastoma cell line (SHSY-5Y). Characterization of liver oil of* Echinorhinus brucus* revealed the presence of palmitic acid (15%), oleic acid (12%), stearic acid (8%), docosahexaenoic acid (DHA) (18%), and eicosapentaenoic acid (EPA) (16%). It was also found to be a good source of squalene (38.5%) and fat soluble vitamins such as A, D, and K (vitamin A: 17.08 mg/100 g of oil, vitamin D: 15.04 mg/100 g oil, and vitamin K: 11.45 mg/100 g oil). Since it was found to be rich in essential fatty acids, fat soluble vitamins, and squalene, it can be considered as better dietary supplement. The oil of* Echinorhinus brucus* also showed high* in vitro* cytotoxic effect against the human neuroblastoma cell line (SHSY-5Y) and the IC_50_ value laid between 35 and 45 ng.

## 1. Introduction

The immense health benefits of the shark liver oil have been studied in the past few decades and were reported as a source of nutrients such as lipid soluble vitamins and essential fatty acids. Because of its therapeutic health benefits such as wound healing property and the stimulation of haematopoiesis, it is being used in traditional medicine in Scandinavian countries [1]. The high content of *ω*-3 polyunsaturated fatty acids also contributes to its multifunctional properties. It has been confirmed that these *ω*-3 PUFA retard the formation of clots responsible for cardiovascular diseases. There is a close relationship between the consumption of the oil and the decrease of cholesterol and triglyceride level in the blood, together with a decrease in blood pressure, factors that reduce the risk of suffering coronary atheromatosis [2] and have been used in rate of sharks being caught during deep sea fishing in India at the time of fishing trawls which are of significant numbers. A variety of valuable products directly from shark include surface active agents, aromatic, lubricants, artificial silk, cosmetics, and pharmaceuticals [3]. The low reproductive capability and slow growth rate of sharks make it impossible for its liver oil commercialization in India [4]. The consumption of fish oil got its high interest in the past couple of years due to its varied health benefits [5]. Shark liver oil contains a group of ether-linked glycerol known as 1-O-alkylglycerols, n-3 PUFA, squalene, and some vitamins. These components have been reported to present multiple biologic activities, including inhibition of tumor growth [6, 7] and enhancement of both macrophage activation [8] and specific immunity in rodents and humans [9]. Because of its dual content of n-3 PUFA and alkylglycerols, shark liver oil is of particular interest in nutrition. The liver oil extracted from these sharks was used in treatment of various diseased conditions like infectious diseases and cancer [10]. Shark liver oil is found to be rich in various vitamins like vitamin A and tocopherol [11]. The oil extracted from deep sea sharks contains a towering level of PUFA, squalene, alkylglycerols, and trivial levels of free fatty acid, sterol, pristane, and wax ester [12]. Certain bioactive compounds, namely, alkyl glycerol and their derivatives, have established influence on many physiological mechanisms in the human body. Antiproliferative effect of alkyl glycerol against colon cancer cells has already been reported [13]. Squalene and alkyl glycerol and some fatty acids have been reported to have anticarcinogenic effect against mammalian carcinoma. The western diet contains disproportionately high levels of *ω*-6 and low levels of *ω*-3 fatty acids, resulting in high *ω*-6/*ω*-3 ratios that are linked to multiple pathological conditions. Animal and human studies suggested that decreasing *ω*-6/*ω*-3 ratio ameliorates cardiovascular disease and improves body's other metabolic outcomes [14]. Recently there has been a keen interest in exploring the role of *ω*-3 FAs in several diseased conditions [15]. Although the efficacy of *ω*-3 FAs in human cancer remains inconclusive,* in vitro* and* in vivo* animal studies suggest that *ω*-3 FAs may have a protective effect against breast, prostate, liver, colon, and skin cancer in addition to neuroblastoma [16–20]. Neuroblastoma (NB) is one among the most common extra cranial solid cancers in children and the most common one during infancy with a very high severity. When it comes to the cancer treatment, chemotherapy is the most employed method of treatment for cancers in addition to radioactive therapy, but they have numerous side effects and hence the search for an alternative medicine with no or little side effect is a prerequisite.* Echinorhinus brucus* are deep sea sharks found mainly in the eastern Pacific Ocean apart from there; it is also found in the tropical and temperate waters worldwide. This rarely encountered shark swims close to the bottom of the sea floor, typically at depths of 400–900 m (1,300–3,000 ft), though it may enter much shallower water. Its liver oil is highly valued in countries like South Africa as medicine, whereas in India the oil is considered poor and is used to coat canoes to discourage wood boring beetles. Our current study is to evaluate the cytotoxic activity of the liver oil extracted from* Echinorhinus brucus* against neuroblastoma cell lines and also to profile its biochemical constituents.

## 2. Materials and Method

### 2.1. Sample Collection and Oil Extraction

Bramble shark (*Echinorhinus brucus*) (12 kg and 5.5 ft) was caught during cruise number 318 (400–600 m) on the Fishery Oceanographic Research Vessel (FORV) Sagar Sampada between Mangalore and Kochi on the west coast of India. Deep sea trawl nets were used for fishing and samples were immediately frozen at −20°C onboard and subsequently brought to the laboratory for further analysis. The liver (3.5 kg) of bramble sharks collected was excised and weighed. Lipid extraction was done as described by Folch et al. [21], employing a 2 : 1 mixture of chloroform-methanol to a weighed portion of the liver sample. The oil was stored in amber colored bottles, under nitrogen, at −60°C.

### 2.2. Fatty Acid Analysis

Fatty acid methyl esters (FAMEs) were analyzed by the method of Sankar et al. [22]. A fraction of the lipid extract was saponified with 0.5 N NaOH in methanol followed by methylation in 14% boron trifluoride in methanol (BF_3_/MeOH). Methyl esters of the fatty acids thus obtained were separated by gas chromatography (Thermo Trace GC Ultra) equipped with a Perkin Elmer Elite 225® capillary column (30 m × 0.25 mm × 0.25 *μ*m) and a flame ionization detector. The carrier gas was nitrogen and the flow rate was 0.7 mL/min. The initial temperature of column oven was set as 110°C which increased at the rate of 2.7°C/min until 240°C, followed by a hold for 4 min. This resulted in a total run time of 57.15 min. Injector and detector temperature was kept at 300°C. Hydrogen and air flow rates were adjusted to 45 mL/min and 450 mL/min, respectively. The individual components were identified by comparing retention times with those obtained from the FAME mixture standard (Supelco-Sigma Aldrich QumHmica, Mexico). Quantitative composition was calculated on the basis of the percentages of a specific peak area to the total peaks area. Quantitative data were corrected for differences in detector responses through analysis of authentic standards of each reported fatty acid. All solvents used were analytical reagent grade from Merck (Merck-Mexico, S.A).

### 2.3. GC-MS Quantification of Squalene

GC-MS (Perkin Elmer Turbomass MS and Perkin Elmer Autosystem XL GC) method was employed to identify and quantify squalene from shark liver oil sample. Squalene is derived from shark oil,* Echinorhinus brucus*, through a series of processes, namely, oil extraction and separation of nonsaponifiable matter by the method of Folch et al. [21]. From the nonsaponifiable matter squalene is identified and quantified by GC-MS method using commercial squalene (Sigma) standard. A concentration of 20 micro litters was used as standard.

### 2.4. Fat Soluble Vitamin Extraction and Quantification by Reverse Phase HPLC Method

Fat soluble vitamins, namely, vitamin A, vitamin D2, and vitamin K2, were identified and quantified by reverse phase HPLC method from shark liver oil extracted from* Echinorhinus brucus*. Nonsaponifiable matter was separated from shark liver oil by the method of Folch et al. [21]. Fat soluble vitamins were identified from the extracted nonsaponifiable matter and quantified by reverse phase HPLC using commercial standard.

### 2.5. Cell Culture

Cell culture SH-SY5Y cells (ATCC, USA) were routinely cultured in Dulbecco's Modified Eagle Medium (DMEM) (Invitrogen Inc., USA) supplemented with 10% fetal bovine serum (FBS), 100 unit/mL penicillin, and 100 *μ*g/mL streptomycin at 37°C with 5% CO_2_.

### 2.6. Cytotoxic Effect of Liver Oil Using MTT Assay

The viability of cells was assessed by MTT assay using cancer cell lines. The assay is based on the reduction of soluble yellow tetrazolium salt to insoluble purple formazan crystals by metabolically active cells. Only live cells are able to take up the tetrazolium salt. The enzyme (succinate dehydrogenase) present in the mitochondria of the live cells is able to convert internalized tetrazolium salt to formazan crystals, which are purple in color. Then the cells are lysed and dissolved in DMSO solution. The color developed is then determined in an ELISA reader at 570 nm. Briefly, the MTT solution (5 g/L) was added to each well and incubated at 37°C for 4 h. After the removal of culture medium, 100 *μ*L dimethyl sulfoxide was added to each well to dissolve the formazan. The optical density was measured at 570 nm using a microplate reader (Tecan, Austria). The optical density of the control group was considered as 100% of the cell viability. Cytotoxic effect was evaluated in SHSY-5Y cell line in different concentrations, 0.01 ng to 100000 ng.

## 3. Results and Discussion

### 3.1. Fatty Acid Analysis

Some fatty acids were identified in the saponifiable fraction of shark liver oil in this study and summarized in Table 1. Major nutritionally significant fatty acids were observed in the profile and they contribute to the bioactivity of corresponding oil. Palmitic acid (14.79 ± 0.03), myristic acid (2.36 ± 0.40), and stearic acid (8.27 ± 0.20) are the prominent saturated fatty acids. As unsaturated fatty acids are taken into account oleic acid (12.13 ± 0.11), linoleic acid (9.24 ± 0.05), linolenic acid (2.23 ± 0.13), eicosapentaenoic acid (EPA), and docosahexaenoic acid (DHA) contribute the major portion (16.27 ± 0.22 and 18.1 ± 0.99, resp.) (Table 1). This is in accordance with previous results [23]. Diet composition and the water temperature may be two important factors responsible for the low content of EPA and DHA in the liver oil of sharks, as well as the fact that planktonic crustaceans are an important source of food for them, and its EPA and DHA concentration is affected by the environmental temperature in this sense: an increased in water temperature could cause a decrease in the EPA and DHA levels in shark liver oil [24]. Lipid content and composition in shark liver oil could be affected by different known factors, such as fishing season, species, location, and availability of food. Shark liver oil is regarded as an excellent source of polyunsaturated fatty acids (PUFA). Two of these acids, eicosapentaenoic acid (EPA) and docosahexaenoic acid (DHA), have been reported to be important in preventing or reducing heart diseases [25] and inflammatory disorders and as a nutritional supplement for the brain and retina development in babies. Saturated fatty acids (SFA), such as oleic, palmitic, and stearic acids, are used as energy source, building blocks for structural elements, for protein modification, and for regulation of gene transcription. Compositional analyses have shown remarkable specificities for particular SFA in cellular compartments, though the metabolic aspects and health effects of the individual SFA are hard to examine. Adipose tissue and liver own the capacity to* de novo* synthesize and store SFA, particularly palmitate (16:0), from polar precursors, notably glucose. In addition to 16:0, the mammary gland owns the means to produce other specific SFA, such as myristic (14:0) and lauric (12:0) acids, providing a source of easily available energy and microbial protection to ensure the growth, development, and survival of the mammalian offspring [26].

### 3.2. GC-MS Quantification of Squalene

**Figure 1:**
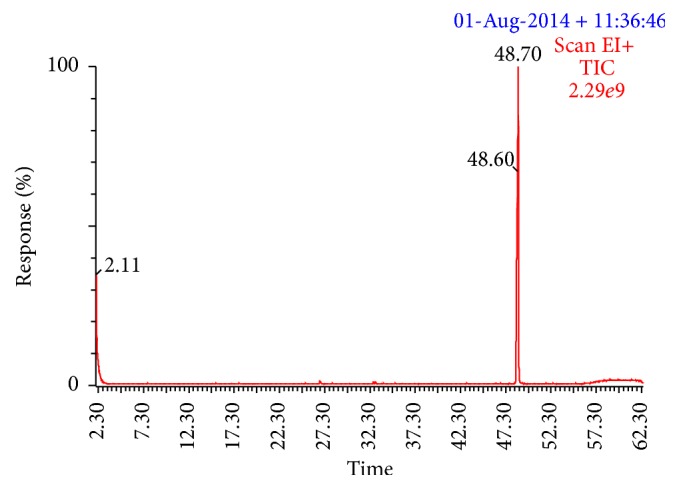
GC-MS chromatogram of squalene standard (20 *μ*L). Showing its response at the 49th minute.

**Figure 2:**
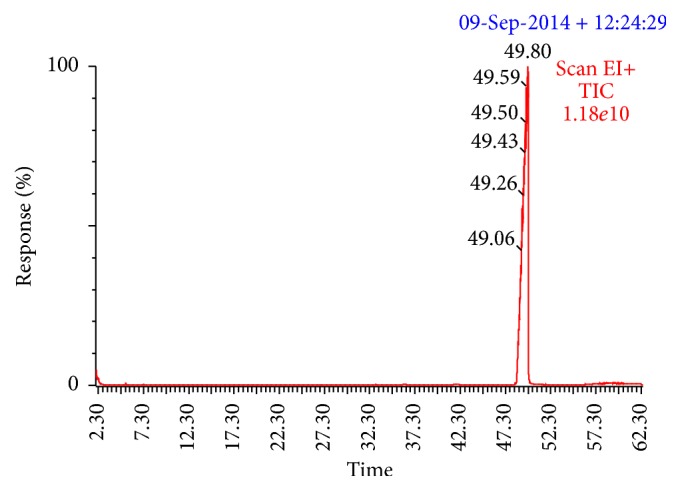
GC-MS chromatogram for squalene extracted from liver oil of* Echinorhinus brucus*.

It is known that shark liver oil is recommended as a nutritional supplement because of its high content of hydrocarbon squalene. Squalene, a C_30_H_50_ triterpenic hydrocarbon with six nonconjugated double bonds, is usually found in the deep sea shark, olive oil, and palm oil [27]. It is a known natural antioxidant that plays an important role in lowering blood cholesterol, enhancing the antitumor action of chemotherapeutic agents, inhibiting cancer growth, and increasing the efficiency of the immune system [27]. Consequently, the presence of squalene in brucus oil would improve its nutraceutical value. Squalene standard peak is showed in Figure 1. Squalene concentration in the shark liver oil was calculated to be 38.4% (Figure 2). The obtained result has good accordance with work done by Bakes and Nichols, 1995 [28]. Squalene is proven to be involved in the function of liver as a hydrostatic organ [12]. The amount of squalene found in the liver from* E*.* brucus* analyzed in this study was higher than that found by Deprez et al. [12]. The differences in the amount of squalene may be due to a variety of factors; seasonal variation and within-species difference in oil composition have previously been documented [29].

### 3.3. Chromatogram of Fat Soluble Vitamins

**Figure 3:**
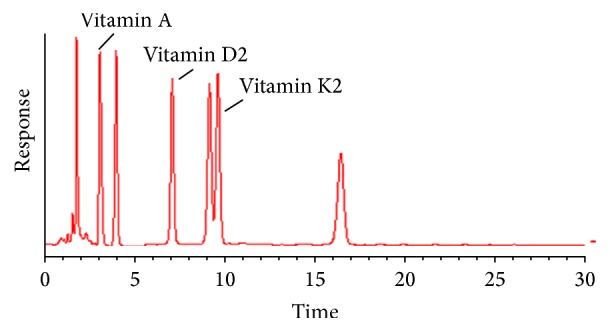
HPLC chromatogram of fat soluble vitamins extracted by reverse phase HPLC method. Quantitative data were corrected for differences in detector responses through analysis of authentic standards of each reported vitamin.

Fat soluble vitamins, namely, vitamin D2 and vitamin K, were identified and quantified by reverse phase HPLC method from shark liver oil extracted from* Echinorhinus brucus*. The column is a reversed-phase silica column with an embedded sulfonamide polar group to enhance the stationary phase. This column has selectivity similar to a C18 column for analytes of low polarity, with the added advantage of compatibility with aqueous only mobile phases. Some classes of compounds (e.g., nitroaromatics) show significantly different selectivity patterns on this bonded phase. A corresponding peak of vitamins A, D, and K was observed at the 3rd, 7th, and 9th minutes. The level of vitamin K2 and vitamin D2 was found to be 11.45 mg/100 g oil and 15.04 mg/100 g oil, respectively, whereas vitamin A level was found to be 17.08 mg/100 g of oil (Figure 3). Quantitative composition was calculated on the basis of the percentages of a specific peak area to the total peaks area. Quantitative data were corrected for differences in detector responses through analysis of authentic standards of each reported vitamin. Health problems can prevent your body from absorbing vitamin K, such as gallbladder or biliary disease, cystic fibrosis, celiac disease, and Crohn's disease. During the past decade, major advances have been made in vitamin D research that transcends the simple concept that vitamin D is important for the prevention of rickets in children and has little physiologic relevance for adults. Inadequate vitamin D, in addition to causing rickets, prevents children from attaining their genetically programmed peak bone mass, contributes to and exacerbates osteoporosis in adults, and causes the often painful bone disease osteomalacia. Patients who suffered from vision reduction in semidarkness conditions (nyctalopia or night blindness disease) were cured by topical application of liver juice or ox liver extract (previously cooked) in the eye. About this procedure, currently the biological role of vitamin A in the visual cycle is well known and it is directly related to the rod cells present in retina of the eye [30].

### 3.4. Cytotoxic Evaluation of Liver Oil on Neuroblastoma Cell Line

**Figure 4:**
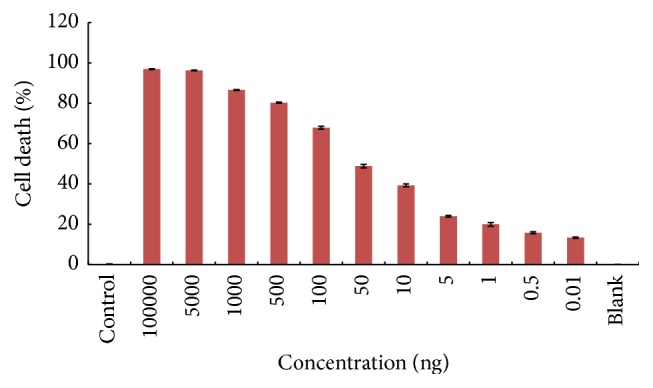
Cytotoxic effect of liver oil using MTT assay. Data expressed as a percentage of cell death. Value represents the mean ± SD.

It was observed that shark liver oil extract from* Echinorhinus brucus* has cytotoxic effect in a dose-dependent manner (Figure 4). The liver oil extract has shown cytotoxic effects against SH-SY5Y cell lines with IC_50_ of 35–45 ng. The cytotoxic effects were analyzed following 48 hours of treatment with the liver oil extract. Our study suggests for the first time that* Echinorhinus brucus* liver oil could play a role in developing new strategies for prevention and treatment of neuroblastoma. Related studies show that squalene, alkyl glycerol, and some fatty acid present in liver oil exert anticancerous effects [31].

## 4. Conclusion

There was no comprehensive data regarding biochemical characterization of bioactive compounds in deep sea shark liver oil from Indian EEZ. Shark liver oil had a good content of squalene, indicating that it can potentially be used as a nutritional supplement. Shark liver oil contains a higher amount of saturated fatty acids (SFA) and polyunsaturated fatty acids (PUFA). They have multiple* in vitro* and* in vivo* properties: they reduce the side effects of radiotherapy, inhibit tumor growth, and both stimulate and modulate immune responses. Significant cytotoxic activity was noticed for the oil used for this study which provided its potential use against human neuroblastoma and its use as an alternative for the chemotherapy currently being employed against neuroblastoma with severe side effects. We suppose that shark liver oil is a good candidate for further studies in cancer therapy.

## Figures and Tables

**Table 1:** Fatty acids from liver oil of *Echinorhinus brucus*. Data expressed as a percentage of wet weight. Value represents the mean ± SD.

Fatty acid	(%) in terms of total fatty acids
C14 (myristic acid)	2.36 (±0.40)
C16 (palmitic acid)	14.79 (±0.03)
C16:1 (palmitoleic acid)	3.51 (±0.01)
C17:1 (heptadecanoic acid)	2.01 (±0.06)
C18:0 (stearic acid)	8.27 (±0.20)
C18:1n9 (oleic acid)	12.13 (±0.11)
C18:2n6 (linoleic acid)	9.24 (±0.05)
C18:3n3 (*α*-linolenic acid)	0.89 (±0.09)
C18:3n6 (*γ*-linolenic acid)	2.23 (±0.13)
C20:1 (eicosenoic acid)	0.55 (±0.02)
C20:3n3 (eicosatrienoic acid)	5.17 (±0.10)
C20:5n3 (EPA)	16.27 (±0.22)
C22:6n3 (DHA)	18.1 (±0.99)
C23:0 (tricosanoic acid)	1.13 (±0.89)
